# Color-Vision and Color-Blindness

**Published:** 1896-12

**Authors:** 


**Color-Vision and Color-Blindness.** By J. Ellis Jennings, M.D. Pp. ix., 115. Philadelphia: The F. A. Davis Company. 1896.

This book begins with an extremely good historical sketch. Soon after Prof. Holmgren had perfected his method of examination by coloured wools, he found that out of 266 employés on the road of the Upsala-Gefle line—about whose vision no suspicion had existed—13 were colour-blind.

Special public attention was called to the subject in 1875 by an enquiry into the causes of a railway accident which had taken place in Sweden, from which it appears that colour-blindness was the cause of the disaster.

Our author having described colour-sensations, goes on to discuss the theories of colour-perception and colour-blindness, classifying the latter defect as total and partial. Total colour-blind persons seem to see the world somewhat as though it were an engraving in black and white; but these cases are extremely rare. It is said that ordinary colour-blind people make excellent engravers from pictures, of which they get but an imperfect conception of the colouring. The dangers to the community of colour-blind employés, particularly on railroads and ships, are considered, and it is explained how impracticable it would be to make any change in the present system of signalling in order to accommodate the colour-blind. The various methods, old and recent, for the detection of colour-blindness are described, with their practical application to candidates for examination by the colour-sense. There is a good chapter on acquired colour-blindness. The book is well printed and is very readable.

